# Biologically Motivated Novel Localization Paradigm by High-Level Multiple Object Recognition in Panoramic Images

**DOI:** 10.1155/2015/465290

**Published:** 2015-09-17

**Authors:** Sungho Kim, Min-Sheob Shim

**Affiliations:** Advanced Visual Intelligence Laboratory, Yeungnam University, 280 Daehak-ro, Gyeongsan, Gyeongbuk 712-749, Republic of Korea

## Abstract

This paper presents the novel paradigm of a global localization method motivated by human visual systems (HVSs). HVSs actively use the information of the object recognition results for self-position localization and for viewing direction. The proposed localization paradigm consisted of three parts: panoramic image acquisition, multiple object recognition, and grid-based localization. Multiple object recognition information from panoramic images is utilized in the localization part. High-level object information was useful not only for global localization, but also for robot-object interactions. The metric global localization (position, viewing direction) was conducted based on the bearing information of recognized objects from just one panoramic image. The feasibility of the novel localization paradigm was validated experimentally.

## 1. Introduction

In the near future, service robots, such as care robots, education robots, and home robots, will be all around us. Imagine a care robot in a home environment. A patient in bed requires drinking a glass of milk from the refrigerator. The patient will command the care robot by saying “Robot, give me a cup of milk from the refrigerator.” The robot has the ability to recognize its current location and go to the refrigerator. The robot will open the door and bring a cup of milk to the patient. Therefore, a service robot needs to recognize its current position in a complex environment and interact with the objects around it. To achieve successful self-localization, the robot should satisfy the following three requirements:

- Global metric localization for the kidnapping problem.
- Fast localization from a single image.
- Robot-object interaction for visual servoing.

A robot should have the ability to determine its global location to successfully handle the self-initialization and kidnapping problem. Several approaches have been proposed to handle such problems. Park et al. proposed a hybrid map of the object and spatial layouts using a stereo camera to localize globally [1]. Angeli et al. suggested a topological visual SLAM (simultaneous localization and mapping) to determine the global localization [2]. Visual words were used to handle the global location, and odometry information was combined to provide metric information. Ramisa et al. also proposed a topological localization method using affine invariant features [3]. Although these approaches can provide global location information, they used additional information, such as stereo and odometry, for global metric localization. An additional requirement is fast global localization capability using just one image frame. Most approaches can achieve topological localization by recognizing objects or scenes from an image [4]. Metric localization is possible if there is a depth cue (stereo camera) or motion cue (structure from motion) [1, 4]. The last requirement is the capability of robot-object interactions for visual servoing. Robots should have object label and position information.

Several paradigms have been proposed for mobile robot localization. Initially, artificial landmark-based approaches were proposed [5, 6]. After then, the SLAM paradigm became a popular approach because it can build a map and localize itself simultaneously using the extended Kalman filter and an invariant feature, such as SIFT [7, 8]. A particle filter-based statistical estimation was also useful in the SLAM approach. These paradigms were partially successful because they could estimate location information relatively accurately by matching low level features, such as corner points or invariant features, in multiframes. On the other hand, the location estimation error can be large if they use only one frame. In addition, those approaches cannot provide high-level information for robot-object interactions. Recently, Anati et al. used the object category detection method in the particle filtering framework to solve localization at the semantic level [9]. But it required dozens of iterations to find the locations.

**Figure 1:**
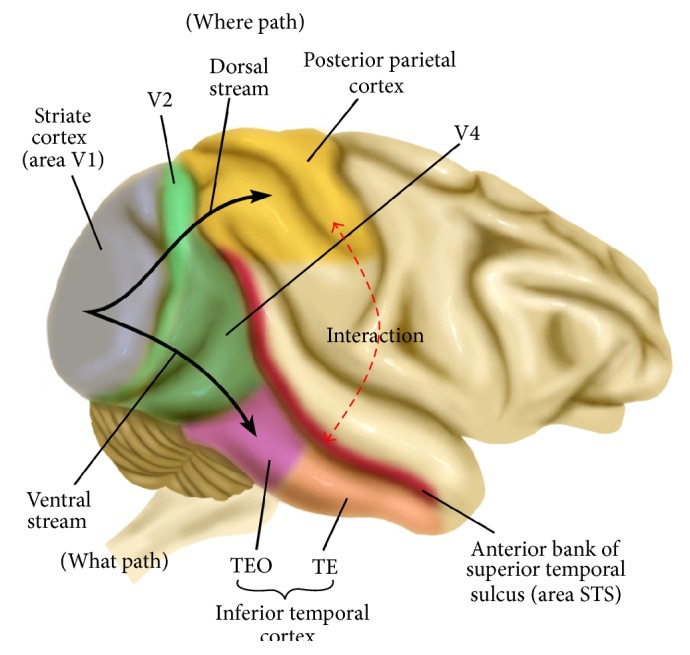
Structure of HVS and the interaction between where path (dorsal stream) and what path (ventral stream).

How can human visual systems (HVSs) localize themselves? HVSs can localize themselves and interact with environment robustly. Do HVSs recognize their locations by point matching, such as SLAM? Most people will say “No.” The localization mechanisms of the HVS were surveyed to obtain the answer or clue. Although accurate mechanisms have not been disclosed, it is evident that object recognition and localization are strongly related according to experimental studies, such as a lesion of the visual cortex (ventral stream and dorsal stream) [10, 11]. As shown in Figure 1, the ventral stream running through the inferotemporal cortex is responsible for visual perception, and the dorsal stream involving the posterior parietal cortex processes the visual information to determine the spatial position. An experiment involving patients (lesion of ventral stream) revealed a long delay in localizing and grasping a target. On the other hand, a second experiment involving patients (lesion of the dorsal stream) showed no perception of an object after movement. This means that object recognition and localization are strongly correlated and that they facilitate each other.

**Figure 2:**
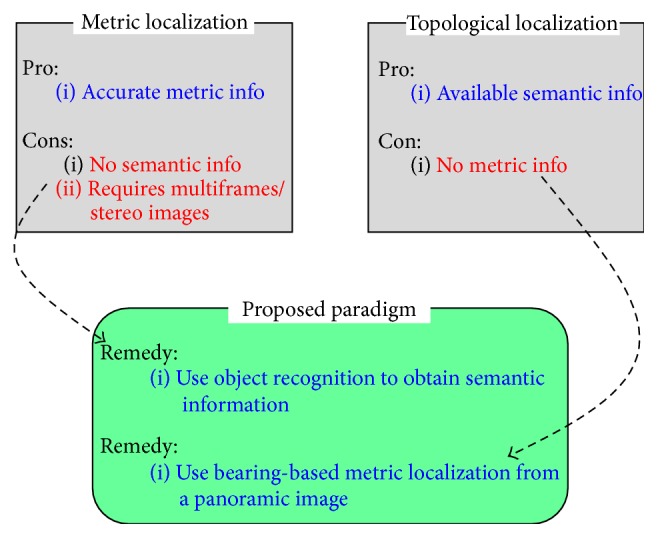
Motivation of the proposed localization paradigm.

Motivated by such biological research results, this paper proposes a novel localization paradigm using only high-level object recognition information from a single image, as shown in Figure 2. The previous feature point matching-based metric localization paradigm can provide accurate location information using multiple frames or stereo images. On the other hand, it cannot provide semantic information, such as the object names. The previous topological localization can provide semantic information with topological location only [12–14]. A novel paradigm is proposed to obtain both the robot location and environmental high-level information to interact with each other. In the proposed paradigm, object recognition information can provide semantic environmental information, and the bearing information of each object can solve the metric localization in a panoramic image. Therefore, the object recognition-centered paradigm can solve the aforementioned problems. The paradigm consists of three parts: omnidirectional panoramic image acquisition, multiple object recognition, and grid-based localization. Multiple object recognition is performed from a panoramic image, and mobile robot localization is conducted using the bearing information of objects. This paradigm can estimate both the spatial position and viewing direction using only one image.

Section 2 overviews the proposed localization system, and Section 3 introduces the omnidirectional panoramic image acquisition camera.  Section 4 explains the multiple object recognition method and Section 5 represents the mobile robot localization algorithm using object information.  Section 6 validates the feasibility of the proposed paradigm experimentally, and Section 7 concludes the paper.

## 2. High-Level Localization Paradigm

**Figure 3:**
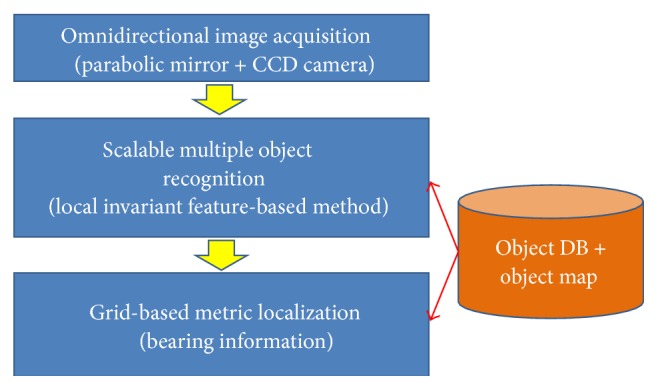
Proposed novel paradigm of localization using high-level object information: given the object database and object-based map, robot location is estimated through the object recognition module and bearing measurement-based localization module.

As shown in Figure 3, the proposed localization system consists of image acquisition, object recognition, and global metric localization. The proposed localization system consists of an offline database construction module and an online localization module. The object database and object-based map are constructed offline. The object DB module contains learned local feature-based object models representing a 3D object as a set of views. Because it is based on a robust invariant feature, the learned models can handle geometrically and photometrically distorted objects in a general environment. The object-based map is built manually by accurately measuring object locations. Online localization was conducted through the panoramic image acquisition module via an omnidirectional camera, multiple object recognition module, and a bearing angle-based localization module. A large field of view is required for object-based localization from a single image. Although there can be several methods for obtaining an omnidirectional image, the parabolic mirror-based panoramic camera was adopted. Details of the camera will be explained in Section 3. After image acquisition, multiple object information (object label and position in image) is extracted by applying the local invariant feature-based method. Object databases (DBs) that have been learned to handle large numbers of objects were used. After such object recognition occurs, the bearing (angle) information of each object can be obtained. The final robot localization (spatial position and viewing direction) is estimated by intersecting the bearing information.

## 3. Panoramic Image Acquisition

**Figure 4:**
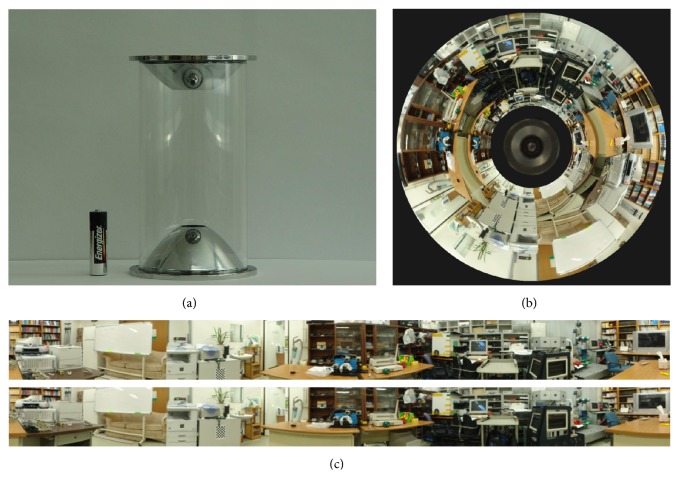
Omnidirectional stereo camera system: (a) parabola mirrors, (b) captured image, and (c) rectified images.

The proposed localization method uses the omnidirectional camera developed by Jang et al. [15].  Figure 4(a) shows the omnidirectional camera system. This is composed of 2 parabolic mirrors and an IEEE 1394 camera (1600 × 1200 image resolution).  Figure 4(b) shows a captured sample image in a laboratory environment.  Figure 4(c) shows the rectified stereo images. Omnidirectional stereo images can be acquired using the camera system. Currently, the upper rectified images are used for the recognizing objects in these images, which can provide higher image resolution than the lower images.

## 4. Multiple Object Recognition

In multiple object recognition module, learned objects stored in the object database can be recognized. Each recognized object can provide an object label and a bearing angle measurement. Because the resolution of a rectified image is 1800 × 161, the bearing measurement resolution of the top-line is 0.2 deg/pixel. A powerful and efficient 3D object representation, learning, and recognition method is introduced. Any 3D object can be represented by a set of multiple views. Each view consists of local features. The sharing concept was applied to the features and views of the scalable object representation. According to recent works, dense and redundant low level features can be reduced by the unsupervised clustering-based feature selection [16]. He and Chen proposed an incremental multiple object learning, recognition, and localization using a multilayer perceptron [17]. Although it is an adaptive object learning framework and works well for an input video stream, it can only localize objects in 2D image space. Li et al. proposed structured subspace learning to reduce the gap between the low level features and semantics in data representation [18]. The previous studies usually focused on 2D objects. This study focused on a scalable 3D object representation.

### 4.1. Scalable 3D Object Representation [19]

Simply storing all possible views of many 3D objects requires huge memory and recognition time. The main cause of this is the redundancy in DB generation. If an object is represented well, the redundancies can be reduced effectively. In advance, a local feature-based object representation, especially common-frame constellation model (CFCM), is adopted instead of holist appearance representation [20]. The CFCM representation scheme provides useful advantages in terms of the computational and redundancy aspects. Because the visual features in a CFCM are conditioned on the camera view, local features are independent of one another. This reduces the computational complexity from *O*(*N* ^2^) to *O*(*N*), where *N* is the number of features. In a CFCM, the source of redundancies can also be found explicitly. One is the object features and the other is the object parameters of the object ID and view point, respectively. Because the training images are composed of many multiple views and objects, there are redundant features and views. These redundancies can be reduced by applying the clustering concept to both features and views.

**Figure 5:**
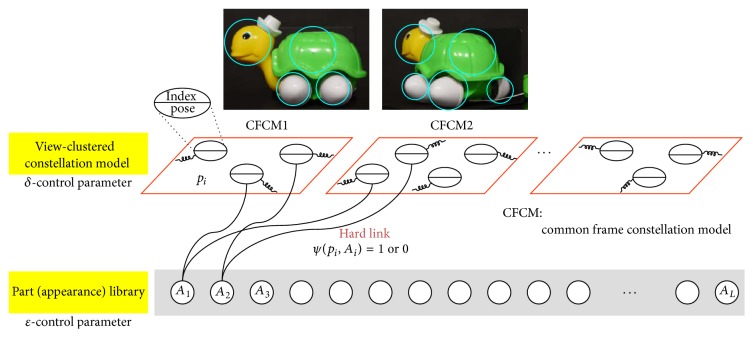
Shared feature-based common frame constellation models (CFCMs) which are view clustered.

Based on these motivations, Figure 5 presents the proposed scalable object representation scheme. The bottom table shows the appearance feature library. Each feature represents an appearance vector obtained via vector clustering. The appearance feature of an individual part is represented by G-RIF (generalized robust invariant feature), which is a generalized version of SIFT and shows better performance than SIFT [21]. A 3D object is represented as a set of view clustered CFCMs. Each CFCM contains object parts that have the part pose and the link indices to appearance libraries. The part pose represents the part size, part orientation, and position in a CFCM. Similarly, each element in the library contains all the links to the parts in CFCMs. This fact can be used to generate hypotheses during object recognition.

### 4.2. Leaning for 3D Object Database

**Figure 6:**
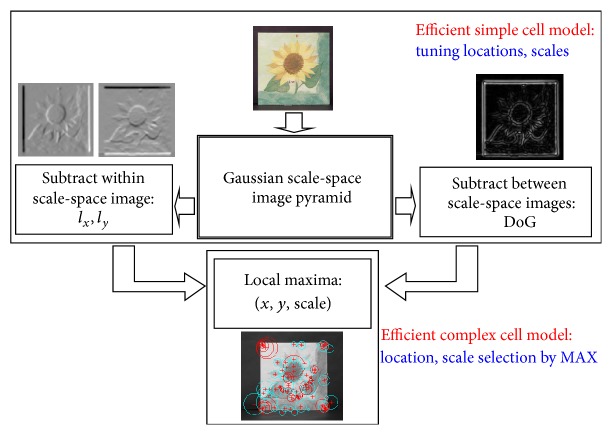
Procedure for the visual part detection: convex and corner parts are detected.

First, an object is decomposed into convex parts and corner parts using Harris corner and DoG (difference of Gaussian) detectors. Second, the part size is determined at the local maxima of convexity where DoG is compared in scale space (see Figure 6). This method can extract complementary object parts. The dominant orientation is calculated using the weighted steerable filter. Finally, the detected convex part is encoded using a set of localized histograms (total 21) of an edge orientation (4 bins), edge density (1 bin), and hue (4 bins). This is a generalized form of contextual descriptor [8]. The feature dimension is 189 (21*∗*(4 + 1 + 4)). More details of implementation and performance can be found elsewhere [21]. This feature is called the generalized robust invariant feature (G-RIF) for its properties.

**Figure 7:**
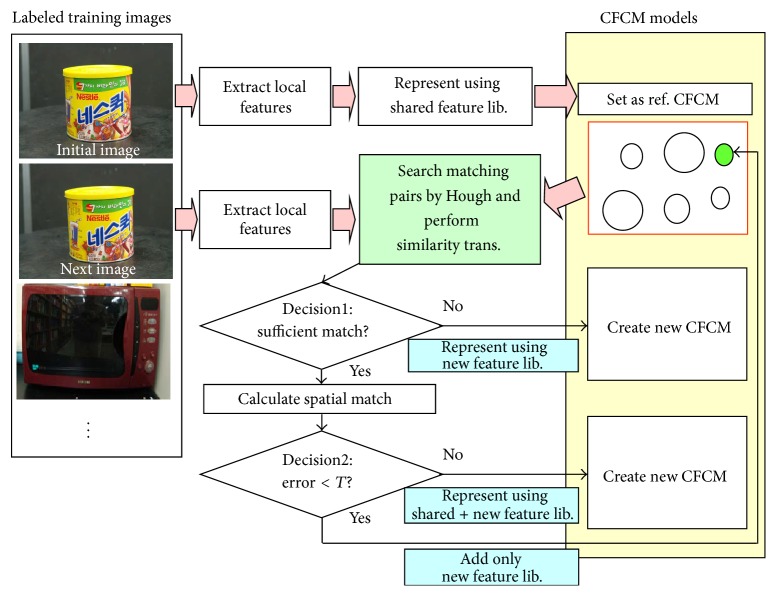
Object learning by sequential view clustering.

As a 3D object is represented by a set of view-tuned CFCMs, the visual parts in a CFCM are conditioned according to the view-tuned parameters. The term view tuned means view clustering in similarity transform space [22].  Figure 7 shows the overall object learning structure. Given the labeled multiviews and multiobjects images, it is important to find view-tuned CFCMs. In a CFCM, each part is represented in terms of a pose and appearance index to the shared feature libraries that were constructed by *k*-means clustering.

Learning is conducted sequentially. The first image is set as a reference CFCM. A CFCM contains an object, view ID, and parts (pose, appearance per part). The pose of a part is obtained directly from the feature detector, and the part appearance is represented using the index of clustered features. The local features are extracted from the next image. Matching pairs are searched between the input feature and CFCM in DB using the Hough transform in pose space (CFCM ID, scale, orientation space). Finally, new CFCMs are constructed according to the following three cases.

**Case 1 .**

If there are few matching pairs (*T*1 < 5), then generate a new CFCM represented in terms of the new feature libraries.

**Case 2 .**

If there are sufficient matching pairs, but the spatial average matching error by similarity transformation is below a predefined threshold (*T*2: usually 4 pixels), then create a new CFCM with a shared feature with new feature libraries.

**Case 3 .**

Finally, if the matching pairs are matched almost correctly, then add new features to the existing CFCM.

**Figure 8:**
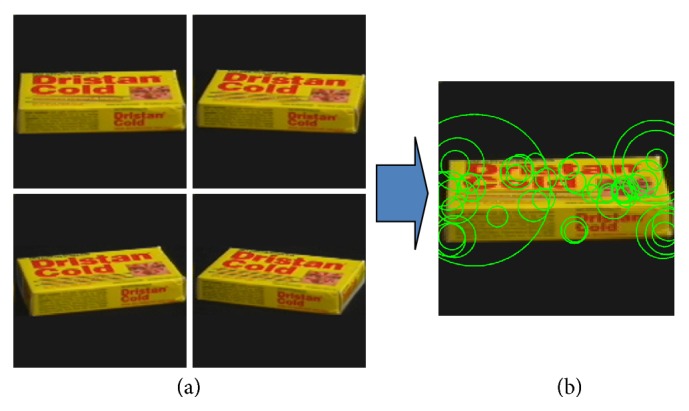
View clustering: (a) training sequence; (b) view-tuned CFCM.

Figure 8 gives an example of sequential CFCM construction results from multiple object views (COIL-100 DB). Many view-tuned CFCM images are obtained from 4 training images. The CFCM construction method can extract the distinguishable multiple views for 3D objects in similarity transform space (affine transformation is not suitable for 3D objects since the feature detector is invariant up to similarity transform). More details about the learning process are explained elsewhere.

### 4.3. Multiple Object Recognition Method

How can we fully utilize the shared feature-based view clustering method in object recognition? Basically, the well-known hypothesis and verification framework is used. On the other hand, it is modified to recognize multiple objects via the proposed object representation scheme.

**Figure 9:**
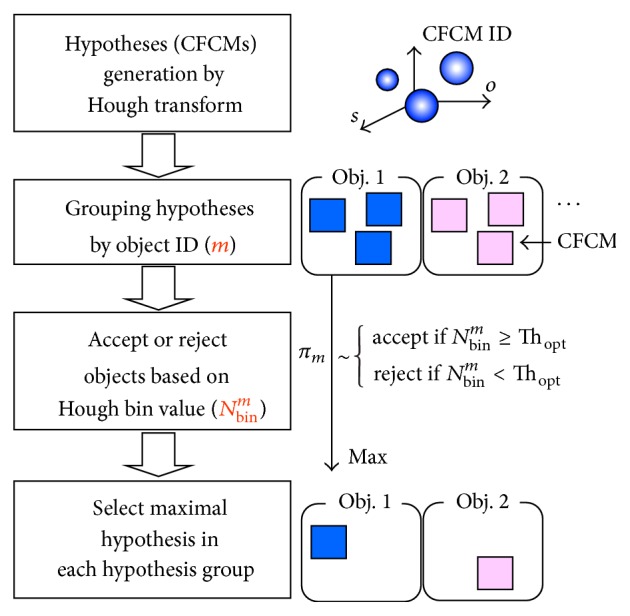
Hypothesis and test (verification) based object recognition procedures.

**Figure 10:**
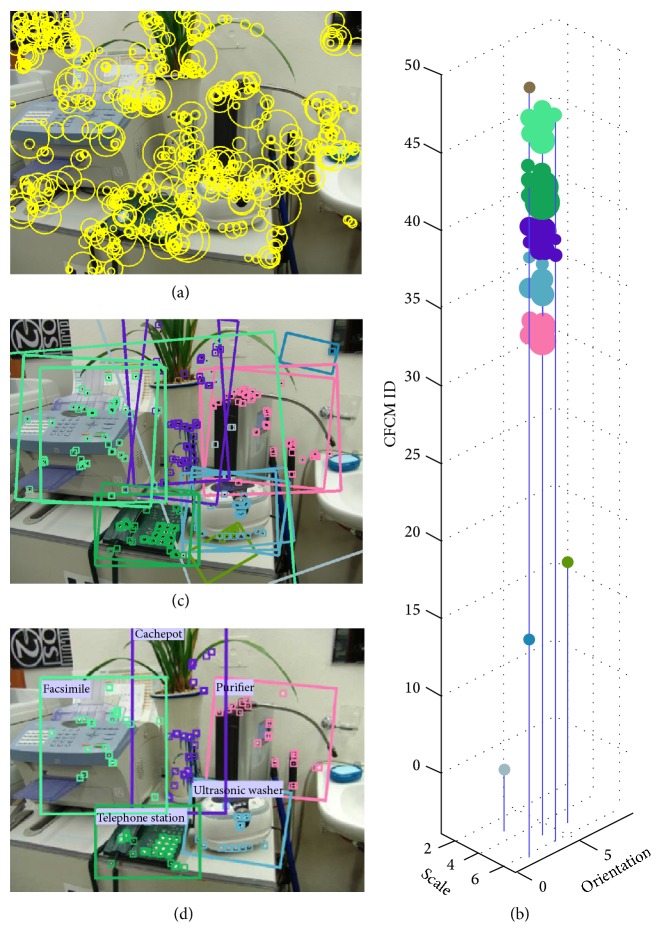
Hypothesis and test based object recognition examples: (a) input image with the detected features, (b) Hough space, (c) hypothesized objects, and (d) final object recognition results.

Figure 9 summarizes the object recognition procedures graphically. All possible matching pairs can be obtained by NN (nearest neighbor) search in the feature library. From these, hypotheses are generated by the generalized Hough transform on a CFCM ID, scale (11 bins), orientation (8 bins) space [8], and grouped by object ID. A decision is made to accept or reject the hypothesized object based on the bin size with an optimal threshold [23]. Finally, the optimal hypotheses that can be best matched to the object features in a scene are selected.  Figure 10 shows the corresponding results.

## 5. Grid-Based Global Localization

**Figure 11:**
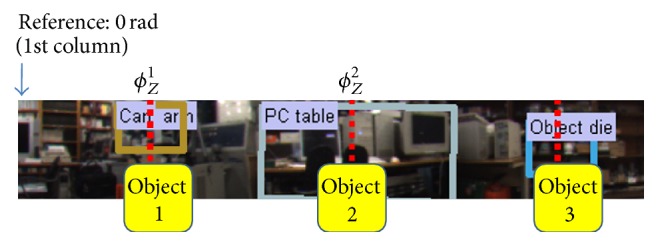
Bearing measurement information of recognized objects in panoramic images.

In the localization module, the recognized object labels are used to achieve data association of objects in a map, and the intersection of bearing measurements is used to accomplish robot localization. Through object recognition, the position of the recognized objects in an image can be estimated. In particular, the column position provides the bearing measurement (*ϕ*_*Z*_^*i*^) of an *i*th object in panoramic images, as shown in Figure 11. In this study, the 1st column of an image is considered as 0 radian. An object center is estimated by the similarity transform of a corresponding CFCM. Given a set of object labels and bearing measurements, the robot localization is defined as the coordinate transformation from reference coordinates to the robot coordinates in 2D space.

**Figure 12:**
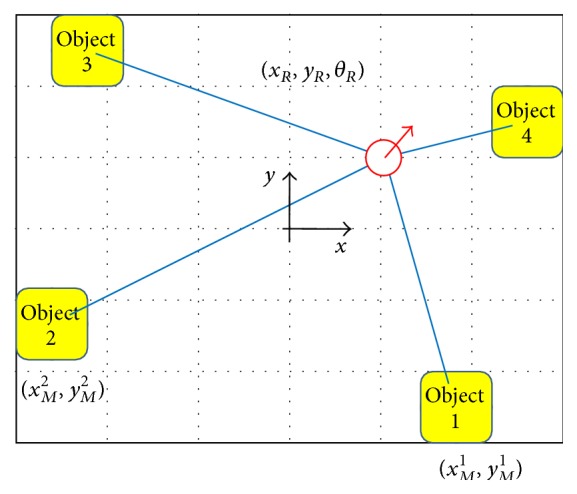
Localization problem is regarded as a coordinate transformation from reference coordinates to the robot coordinates.

Let {*A*}_*Z*_ be a set of bearing measurements by a mobile robot through multiple object recognition; let {*A*}_*R*_ be a set of model bearing measurements after coordinate transformation. The robot localization problem is to estimate *T* = (*x*, *y*, *ϕ*), which is the coordinate transformation function from the reference coordinates to the robot coordinates, as shown in Figure 12. Shimshoni proposed a direct estimation method based on the linear constraints [24]. This method was applied but the estimation results were very unstable due to bearing measurement noise and a small number of measurements (normally 3–6). Fox et al. proposed a Monte Carlo localization method that approximates the posterior by a set of samples [25]. The latter method was also applied, but it takes time to converge. Instead, the grid-based localization method was used. If the coordinate transformation space is divided into a moderate resolution (in current implementation, *δ*_*x*_ = *δ*_*y*_ = 10 cm, *δ*_*ϕ*_ = *π*/180 rad), then the robot location is estimated using (1). *N* denotes the number of recognized objects. If the symbols are specified, then the localization problem is the minimization problem of three dimensions as (2). *ϕ*_*R*_^*i*^ denotes the angle of the model object *i* after a transformation with *T* = (*x*_*R*_, *y*_*R*_, *θ*_*R*_), as shown in (3). The optimal robot location can be obtained using the orientation information by minimizing (2):

(1) T^=minT⁡∑i=1NAZi−ARiT,

(2) x^R,y^R,θ^R=minxR,yR,θR⁡∑i=1NϕZi−ϕRixR,yR,θR,

(3) ϕRixR,yR,θR=tan−1⁡yMi−yRxMi−xR+θR.

## 6. Experimental Results

**Figure 13:**
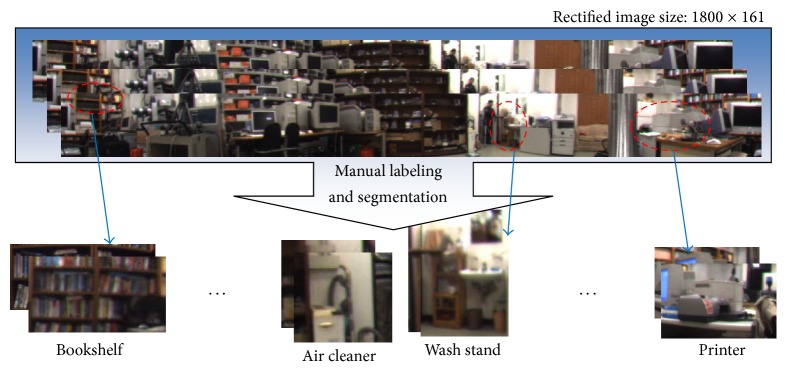
Preparation of object images for learning.

The object recognition-based localization method was applied to a complex laboratory environment.  Figure 13 shows the flow of thee manual object segmentation and labeling process from the rectified images. The images include bookshelf, PC table, air cleaner, wash stand, and printer. Note that the image quality of an individual object is very low.

Table 1 summarizes the dataset for object learning and testing. Every two views were used for object modeling. The total number of objects was 9 with multiple views.  Table 2 lists the results of object learning. Part clustering reduces the size by 44.2%, whereas view clustering reduces the size by 39.8%.

**Figure 14:**
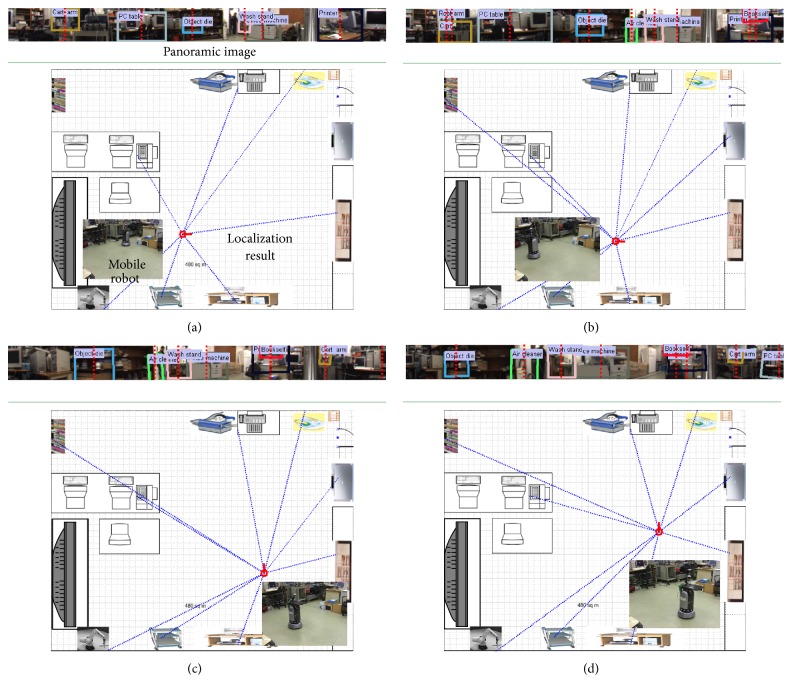
Examples of robot localization using the proposed method: (a) Frame #1, (b) Frame #20, (c) Frame #40, and (d) Frame #60.

Figure 14 shows localization examples of a mobile robot, KASIRI IV, which can move accurately according to the planned path. In each result, the top image shows the recognized objects with object centers that are equal to the bearing measurements. In the bottom image, the red arrow represents the location (position with direction) of the mobile robot, and the data association is linked by the dotted blue line. Note that multiple objects are recognized and used for robot localization.

**Figure 15:**
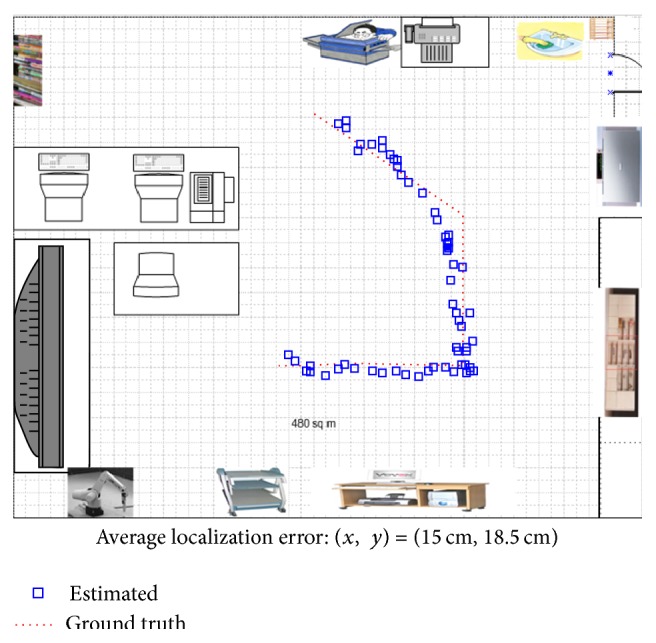
Overall localization performance of the test sequence using the proposed method.

**Figure 16:**
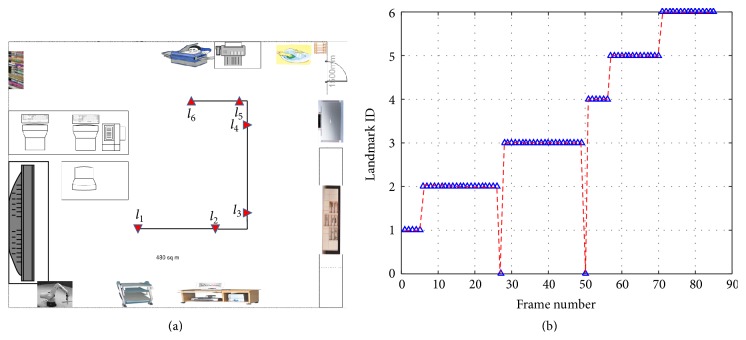
Object recognition-based topological localization: (a) topological map with labels, (b) topological recognition results. Zero label indicates missed object.

Figure 15 summarizes the overall localization performance. The red dotted line represents the true path of the mobile robot and the blue square represents the estimated robot location using the proposed algorithm. The average location error is (*x*, *y*) = (14.5 cm, 18.5 cm), which is relatively large compared to those of the range sensor-based approaches or interesting point-based approaches (normally within 5 cm) in a 10 m × 10 m environment. On the other hand, the proposed system can provide high-level information of an object that is useful for robot-environment interaction. Note that human visual systems (HVSs) can recognize the relative locations with very low metric accuracy but can interact well in an environment with object information. The average processing time of multiple object recognition and metric localization was approximately 20 seconds with a platform of MATLAB R2014b, Intel i7 x990, 16 GB memory. In addition, the proposed semantic recognition-based localization method was compared with the well-known topological localization method [13]. As shown in Figure 16(a), a topological map is generated manually based on the object position information. In this test, the same panoramic images were used and only the recognized object information was checked.  Figure 16(b) shows the topological location results by indicating the landmark ID per frame. The ID with a zero denotes the failed recognition. Although the topological localization showed a high recognition rate (97.6%, 83/85), it could not provide metric localization information.

## 7. Conclusions and Discussion

In this paper, a new robot localization method was proposed using the object recognition method. Instead of fragile low level features, the objects are regarded as natural landmarks for localization. For this system, a multiple object recognition method based on a learned object model and grid-based localization using bearing measurements was introduced. The feasibility of the proposed system was validated experimentally. There are several research directions. Currently, the tracking of objects is not used. If the temporal continuity can be utilized, then a smoother localization can be obtained. In addition, a map is generated manually. Therefore automatic object-map generation should be investigated. The working space can be increased if this can be combined with topological localization.

## Figures and Tables

**Table 1:** Data statistics for learning and test.

Type	Number of scenes	Number of images for 9 objects
Learning	Every two views	128
Test	85	385

**Table 2:** Object learned results.

Type	Before learning	After learning	Compression rate
Number of object images	128	77	39.8%
Number of parts	14,074	7,885	44.2%

## References

[1] Park S., Kim S., Park M., Park S.-K. (2009). Vision-based global localization for mobile robots with hybrid maps of objects and spatial layouts. *Information Sciences*.

[2] Angeli A., Doncieux S., Meyer J.-A., Filliat D. Visual topological slam and global localization.

[3] Ramisa A., Tapus A., López de Mántaras R., Toledo R. Mobile robot localization using panoramic vision and combinations of feature region detectors.

[4] Murillo A. C., Guerrero J. J., Saguüés C. (2007). Topological and metric robot localization through computer vision techniques. *ICRAWorkshop—From Features to Actions: Unifying Perspectives in Computational and Robot Vision*.

[5] Scharstein D., Briggs A. J. (2001). Real-time recognition of self-similar landmarks. *Image and Vision Computing*.

[6] Jang G., Kim S., Lee W., Kweon I. Color landmark based self-localization for indoor mobile robots.

[7] Durrant-Whyte H., Bailey T. (2006). Simultaneous localisation and mapping (slam): part i the essential algorithms. *Robotics and Automation Magazine*.

[8] Lowe D. G. (2004). Distinctive image features from scale-invariant keypoints. *International Journal of Computer Vision*.

[9] Anati R., Scaramuzza D., Derpanis K. G., Daniilidis K. Robot localization using soft object detection.

[10] Himmelbach M., Karnath H.-O. (2005). Dorsal and ventral stream interaction: contributions from optic ataxia. *Journal of Cognitive Neuroscience*.

[11] Blangero A., Coello Y., Striemer C., Rossetti Y., Danckert J., Pisella L. (2008). Dorsal and ventral stream interactions: evidence from optic ataxia. *Brain and Cognition*.

[12] Lin Z., Kim S., Kweon I. S. Recognition-based indoor topological navigation using robust invariant features.

[13] Choi J., Choi M., Nam S. Y., Chung W. K. (2011). Autonomous topological modeling of a home environment and topological localization using a sonar grid map. *Autonomous Robots*.

[14] Kosecka J., Li F. Vision based topological Markov localization.

[15] Jang G., Kim S., Kweon I. (2006). Single-camera panoramic stereo system with single-viewpoint optics. *Optics Letters*.

[16] Li Z., Liu J., Yang Y., Zhou X., Lu H. (2014). Clustering-guided sparse structural learning for unsupervised feature selection. *IEEE Transactions on Knowledge and Data Engineering*.

[17] He H., Chen S. (2008). IMORL: incremental multiple-object recognition and localization. *IEEE Transactions on Neural Networks*.

[18] Li Z., Liu J., Tang J., Lu H. (2015). Robust structured subspace learning for data representation. *IEEE Transactions on Pattern Analysis and Machine Intelligence*.

[19] Kim S., Kweon I. S. (2008). Scalable representation for 3D object recognition using feature sharing and view clustering. *Pattern Recognition*.

[20] Moreels P., Maire M., Perona P. (2004). Recognition by probabilistic hypothesis construction. *Computer Vision—ECCV 2004: 8th European Conference on Computer Vision, Prague, Czech Republic, May 11–14, 2004. Proceedings, Part I*.

[21] Kim S., Yoon K.-J., Kweon I. S. (2008). Object recognition using a generalized robust invariant feature and Gestalt's law of proximity and similarity. *Pattern Recognition*.

[22] Hartley R. I., Zisserman A. (2004). *Multiple View Geometry in Computer Vision*.

[23] Murphy-Chutorian E., Triesch J. Shared features for scalable appearance-based object recognition.

[24] Shimshoni I. (2002). On mobile robot localization from landmark bearings. *IEEE Transactions on Robotics and Automation*.

[25] Fox D., Burgard W., Dellaert F., Thrun S. Monte Carlo localization: efficient position estimation for mobile robots.

